# Developing a model to support safe medication use in the home for foreign-born-persons with language difficulties: a co-creation participatory action research study

**DOI:** 10.1186/s12913-026-15510-1

**Published:** 2026-09-07

**Authors:** Katarina Hjelm, Ulrika Pöder, Anna Ekman, Lisa Hultin

**Affiliations:** 1 Department of Public Health and Caring Sciences, Uppsala University, P O Box 564, Uppsala, S-751 22 Sweden; 2 The Academic Primary Healthcare Centre, Development Unit for Research, Development and Education, Primary Care and Health, Uppsala County Council, Uppsala, Sweden; 3 Uppsala University Hospital, Uppsala, Sweden

**Keywords:** Medication use, Medication safety, Migrants, Language difficulties, Support, Co-creation participatory research design, Patient and public involvement, Relatives, Healthcare personnel, Primary healthcare

## Abstract

**Background:**

Migration has increased population diversity and created challenges for safe medication use. Evidence remains limited regarding factors influencing medication-related problems among migrants and the support needed to optimise medication use in the home. No studies have addressed support for medication use in the home among migrants or proposed models for such support. The aim was to identify the support needed and to develop a model for support to promote safe medication use in the home and prevent medication errors among foreign-born persons with language difficulties.

**Methods:**

A co-creation participatory action research design involved end-users—foreign-born persons and relatives, and staff in healthcare and pharmacies. Focus group discussions, based on previous studies, were analysed using qualitative content analysis and interpreted through Pawson’s framework across four contextual dimensions: individual, interpersonal, institutional, and infrastructural.

**Results:**

The analysis identified seven areas where support was required: routines to support safe medication use; continuity and risk assessment; support for language understanding; resources; information for patients and relatives to understand medication treatment and the prescribing system; staff training; and national measures. Support was needed across all contexts, particularly at the institutional and infrastructural levels through organisational development and clearer guidelines and regulations, but also at the interpersonal level in everyday interactions with healthcare and pharmacy staff. The findings informed the development of a support model anchored in primary healthcare includingan extended interdisciplinary team involving relatives and pharmacists, coordinated by an accessible contact person.

**Conclusion:**

In conclusion, promoting safe medication use in the home for foreign-born persons with limited language proficiency requires placing a well-informed patient at the centre of care; care planning based on individual needs; and coordinated support across individual, interpersonal, institutional, and infrastructural levels. Everyday practice should be critically reviewed to organise teamwork, allow time for risk assessment, and improve patient and relative education. Strengthening continuity, communication, and shared responsibility across a seamless healthcare chain may reduce medication-related risks and support patients and relatives in managing medications safely in the home. Implementation of the developed model should be accompanied by organisational and policy-level changes to enable sustainable, person-centred medication safety.

## Introduction

Migrant health has become a global public health problem due to large-scale international migration. Consequently, many European countries have become multicultural and multilingual societies [1]. Migrants represent a particularly vulnerable group, facing increased risks of morbidity and a higher incidence of non-communicable chronic diseases (e.g. obesity, diabetes, coronary heart disease), which often lead to multimorbidity [2, 3] and polypharmacy, strongly associated with medication errors [4]. Using medications in the home is a complex process, and language difficulties and cultural differences, e.g. in beliefs about illness and medications, can influence adherence to prescribed treatment [5–7]. Many migrants therefore live with complex healthcare needs [2, 3] and may struggle to navigate, access and contact healthcare within often unfamiliar and complex healthcare systems and organisational models [8–10]. At the same time, they may be trying to adapt to life in a new society while experiencing strained living conditions, limited socioeconomic resources, limited social networks and lack of social support [2, 3, 10].

Despite increasing global population diversity, there is limited evidence regarding factors influencing medication-related problems among ethnic minority populations, including migrants, and on the support needed to optimise medication use in the home [7, 11, 12], as well as how such support should be designed. These groups may experience inequities in care safety and face higher risks of patient safety events, including higher rates of adverse drug events and dosing errors compared with the wider population [12]. Furthermore, limited focus has been placed on improving safety for these groups despite international efforts to enhance patient safety and quality of care, thus remaining an under-researched area.

The review of previous literature identified no studies specifically addressing support for medication use in the home among migrants or models for such support. A study exploring healthcare staff perspectives on vulnerable older migrants with cognitive impairment highlighted the need for closer collaboration between healthcare professionals, patients, and relatives to address healthcare system barriers and improve medication safety [5].

Strategies such as pictograms, digital applications and community support may improve medication safety, but their implementation is limited by concerns about accessibility, digital literacy and data protection [13–15]. Research in community pharmacy has also highlighted the importance of culturally responsive communication and support when providing care to migrants and other culturally and linguistically diverse patients [16].

Examples of interventions to prevent medicine-related problems include an Australian Home Medicines Review (HMR) programme evaluated among Chinese and Vietnamese immigrants [17] and a culturally competent education programme in Denmark for Arabic-speaking migrants [18]. The findings indicated confusion about medications and unmet needs of information [17, 18], with a distinct need of HMR but not use of available services [17], and an educational programme [18] that may reduce medication-related problems (MRPs) by providing participants with appropriate knowledge and competencies. Perceived MRPs were concluded likely to improve with increased availability of bilingual and culturally sensitive healthcare providers [17, 18]. However, the outcomes of the interventions are limited by small sample sizes, gender imbalance, diversity in origins and dialects compromising language understanding, and qualitative study designs. Both highlighted the need for further research, consistent with a scoping review reporting limited evidence on facilitators of pharmaceutical care for migrants and refugees [19].

Previously, we studied foreign-born patients [20], relatives [21], and healthcare and pharmacy staff across the healthcare chain [22] to gain a comprehensive understanding of the complex process of medication use in the home among foreign-born persons with language difficulties. Interviews and observations in the home [20] identified two main approaches among patients, some were dependent on others due to language barriers, experienced difficulties accessing primary healthcare and lack of medication information. Others were independent and self-motivated but struggled to access or contact healthcare staff. Language barriers complicated communication between migrants and healthcare services, compromising medication safety.

Relatives supporting persons with limited language proficiency experienced medication management in the home as burdensome, facing high levels of responsibility and insecurity due to lack of information and support, and barriers to communication and understanding [21]. Despite these challenges, relatives were key actors in medication management and expressed a need for accessible, tailored support.

The case study involving healthcare and pharmacy staff [22] revealed challenges in information exchange across the healthcare chain, particularly in communication with prescribing physicians. Additional challenges included limited medication knowledge, differing beliefs about medications, limited understanding of the Swedish prescription and pharmacy system, and the use of generic medications. Suggested improvements included better access to supportive information tools, consistent use of interpreters, and access to bilingual staff.

The next step, and the aim of the present study, is to identify the support needed and develop a model for support to promote safe medication use in the home and prevent medication errors for foreign-born persons with language difficulties. The model will be based on results from a co-creation participatory research study [23]. By addressing multiple dimensions of medication use in the home, communication within the healthcare chain, and the organisation of supportive care, this project contributes to a research field that remains under-studied. Nonetheless, it is essential for ensuring participation and equality in multicultural and multilingual environments [11, 12].

Research on healthcare provided outside hospitals, particularly community-based care for foreign-born persons, remains limited. Such research is essential to develop high-quality, safe and cost-effective care [24, 25]. The need to strengthen care structures outside hospital settings is also emphasised in ongoing reforms aimed at creating a more accessible and equitable primary care system [24]. Analyses of how healthcare systems can improve effectiveness and use resources efficiently conclude that the value of healthcare is developed primarily through interactions between patients and healthcare professionals [24, 25]. A key prerequisite for efficient, high-quality care is the active involvement of patients and their relatives in both the healthcare process and the development of the healthcare organisations. This perspective underpins the design of the present study, which applies a co-creation participatory research approach [23, 26]. By involving relevant stakeholders, the study aims to advance knowledge in community-based healthcare, an area of growing importance for the organisation and delivery of care in the future.

### The Swedish context

Approximately 20% of the Swedish population is foreign-born, comprising people from more than 140 nationalities [27]. Thus, Sweden has a culturally and linguistically diverse population. Swedish legislation [28] stipulates that healthcare should be provided on equal terms to all residents, irrespective of nationality, and individuals who do not speak Swedish are entitled to an interpreter when receiving healthcare [29].

The Swedish healthcare system is tax-funded and decentralised, with 21 regions, and 290 municipalities. Regions are responsible for primary and hospital care, whereas municipalities provide elderly care and home healthcare. Primary healthcare constitutes the foundation of the healthcare system and provides first-contact care through primary healthcare centres, where general practitioners, nurses, and other health professionals provide assessment, treatment, preventive care, and chronic disease management. Patients requiring specialised care are referred to hospitals, with university hospitals providing the most highly specialized services. The organization of primary healthcare varies across regions, although multi-disciplinary team-based care is increasingly emphasised, with person-centred care and patient participation as central principles [24, 25].

Pharmacies constitute an important interface between patients and the healthcare system. They are primarily privately operated but subject to national regulation, providing prescription and over-the-counter medicines, medication counselling, dispensing, generic substitution, and information about patients’ prescriptions. A list over current prescriptions and remaining prescribed medication is offered the patient after visiting the pharmacy. Most prescriptions are electronic and can be dispensed at any pharmacy in Sweden. Furthermore, medication information is shared electronically, enabling authorised healthcare professionals to access patients’ medication information and documentation of prescribed medications in a medication list [30].

Sweden has national high-cost protection schemes for both healthcare visits and prescription medicines. Pharmacies are required to offer the least expensive equivalent generic medicine when available, although patients may choose the original product by paying the price difference. Dose dispensing and support with medication administration may also be provided to patients who have difficulties managing their medicines independently, including through municipal home healthcare [30].

The physician is primarily responsible for the annual review of patient’s medications. According to the legislation, a clinical pharmacist may conduct the review but the physician retains ultimate responsibility. The medication list is provided to the patient by primary or hospital care [30].

## Methods

### Design

**Fig. 1:**
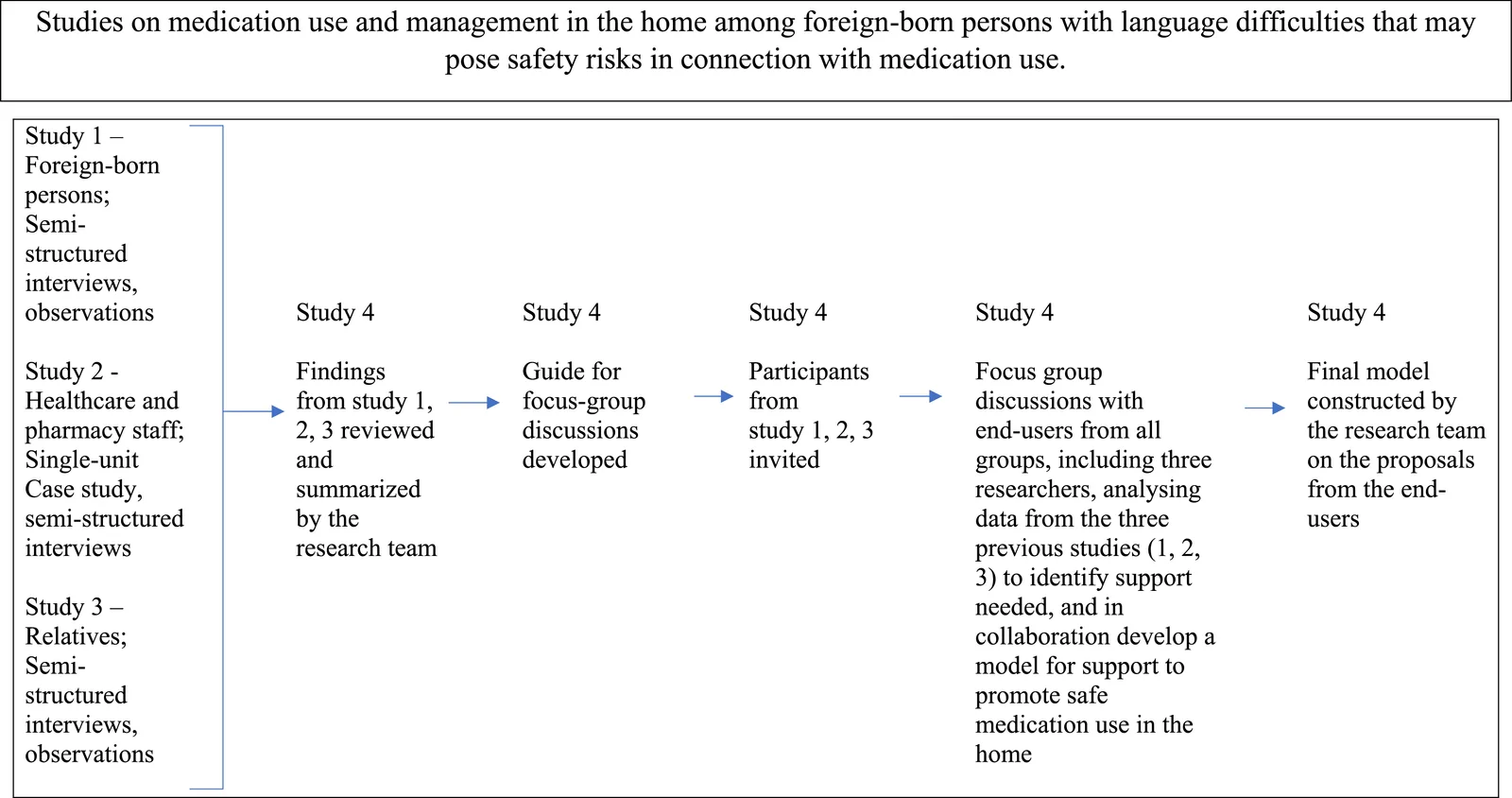
Flow-chart describing the research process from data collection in the three original studies, through extraction of main findings, development of discussion guide, invitation of participants, and focus-group discussions involving end-users, including researchers, to construction of final model for support to promote safe medication use in the home

The study was designed as a co-creation participatory action research process with end-users actively involved in developing a joint model [23] for support. Collaborative work, particularly engaging patients and others involved in care throughout the research process, enables health services to be adapted to the specific context, thereby increasing the acceptability and feasibility of support measures. Shared decision-making in designing new healthcare environments can also help improve the quality of care [23, 26]. In this study, participants collaborated according to the principle of Patient and Public Involvement (PPI) [26] in the data collection (focus group discussions) and data analysis, based on findings from three previous studies in the research project (see Fig. 1). The Guidance for Reporting Involvement of Patients and the Public, GRIPP 2 SF; [31] was followed.

Focus group discussions were held in which results from three previous studies [20–22] on medication use and management in the home among foreign-born persons with language difficulties that may pose safety risks in connection with medication use were presented using PowerPoint with printed handouts. Thereafter, the findings were discussed to identify participant resonance with findings, establish priorities for change, and generate solutions. The previous studies also involved end-users: foreign-born persons and relatives, and staff in healthcare and pharmacies. Focus group discussions were chosen to encourage participant engagement and gain deeper insights into beliefs and experiences, and are considered appropriate for verbalisation and identification of different perspectives and ideas [32]. Such discussions have the strength of stimulating dialogue in which different perspectives become visible and can be openly discussed [32]. Group interaction may also encourage participants to disclose behaviours/attitudes they might not reveal in individual interviews, as the group setting can create a greater sense of security and anonymity. Participants may also feel more comfortable sharing their views when others share similar opinions or when they become engaged in the discussions.

### Setting

End-users collaborating in the co-creation participatory action research process met in a familiar setting: a primary healthcare centre, including municipal home care services, and two pharmacies situated in an immigrant-dense area of a city (about 60%, including second-generation immigrants) (for further details, see [20]. The guiding principle was Patient and Public involvement (PPI), which occurs when patients and members of the public *work directly with* researchers and healthcare professionals as innovators and informants [26]. Five key concepts guided the work: (1) sharing power to achieve a joint understanding; (2) including all perspectives and skills; (3) respecting and valuing the knowledge of all participants as equally important; (4) reciprocity, where everyone benefits from collaboration, and (5) building and maintaining relationships, with particular emphasis on sharing power.

## Participants

Thirteen individuals participated. Ten represented end-users: foreign-born persons and relatives, and staff in healthcare (a primary healthcare centre with connected home care) and pharmacies. They had been involved in previous studies on medication use and management in the home among foreign-born persons with language difficulties that may pose safety risks in connection with medication use [20–22]. They were recruited by staff at the participating organisations and had consented to be contacted for follow-up. The principal investigator (LH) contacted participants by telephone or e-mail to arrange thefocus group discussions. Furthermore, three members from the research team participated and also acted as facilitators and observers: two nurses (LH, KH) with extensive experience in home care, primary healthcare and migration and health, and a clinical pharmacist (AE) with long experience working in pharmacies in immigrant-dense areas. Overall, participants included patients, relatives, nurses, physicians and pharmacists. They were aged between 23 and 73 years (median 52 years), originating from Eritrea, Former Yugoslavia, Iran, Lebanon, Syria, and Sweden.

### Data collection

Data were collected between March 2025 and January 2026. Findings from three previous studies involving end-users: foreign-born persons and relatives, and staff in healthcare and pharmacies were reviewed by the research team [20–22] on what challenges and problems were experienced, what support is needed, and how it should be designed. The findings were summarised and presented digitally using PowerPoint and in printed form. A topic guide was then developed to structure the focus group discussions [23, 32, 33] including the broad, open-ended questions: How should the results be interpreted? How could this be solved? What support could be developed? Is there anything not identified in these three studies that should be added? (See Additional File 1)

Four focus groups, including two to seven participants in each, were held in private conference rooms, mainly outside the primary healthcare centre, and in one case digitally. Comfortable seating, round-table placements, and refreshments were offered to create a relaxed environment to promote group interaction [32].

The groups were moderated by a nurse (LH). An assistant moderator, a nurse or clinical pharmacist (KH or AE), acted as observer and took field notes on interaction patterns, group dynamics, participation levels, impressions of the topics discussed, and non-verbal communication [32]. Both served as skilled facilitators who actively participated in the focus group discussions and the co-creation process. Skilled facilitators play a critical role in guiding discussions and ensuring equitable participation, particularly among vulnerable or marginalised groups [23, 33, 34], such as the migrants in this study.

The first focus group served as a pilot test to refine the topic guide, clarify the moderator’s role and evaluate the overall study approach [32]. As the discussion worked well and the data were of good quality, no corrections were required, and the data were included in the analysis.

Each focus group discussion lasted between 45 and 90 min in free-flowing discussions, with lively interaction and supportive communication, no signs of participant dominance, all contributed equally, and body language demonstrated active engagement [32]. Open and inclusive communication, constructive dialogue, and consideration of different perspectives are important in co-creation participatory action research [23, 33, 34]. Thus, the ‘everyone included’ philosophy, which is the core of PPI, was applied [26]. When needed (*n* = 1), a professional authorised interpreter was present and interpreted literally, using the first person (I-form), remaining neutral and maintaining confidentiality [35]). The focus group discussions were digitally recorded and transcribed verbatim by a professional secretary. The texts were coherent (comprising 108 A4 pages, with 1.5 line spacing) and of high quality.

### Data analysis

Data were analysed using a method described for focus groups [32]. Openness to variation and diversity in the data, and searching for patterns, regularities and contradictions through constant comparison of participants’ statements was emphasised. All transcripts were read several times to gain an overall sense of the data. Text and phrases related to the study aim were identified; meaning units were marked in the transcripts and thereafter coded inductively. Codes were compared for similarities and differences. Groups of codes with shared attributes were organised into sub-categories and categories (labelled as close to the text as possible). Support measures proposed were then sorted deductively into main analytical categories guided by Pawson’s theoretical framework [36], focusing on the science of evaluation and the dimensions of context. The framework describes four contexts: individual, interpersonal, institutional and infrastructural used for evaluation in healthcare (see Table 1). Understanding context (conditions, structures, and environments influencing) is central to realist evaluation to cover important aspects of clinical reality to describe what might work, in what aspects, for whom and how, in the complex healthcare system. A combination of different analytical approaches, inductive and deductive, as described by Mayring [37], was used. The analyses proceeded until no new information emerged, and three focus groups are generally sufficient to achieve this [32], as judged here.

**Table 1:** Pawson’s (2013) evaluation framework describing four contextual dimensions used in the analysis

Context	Content
Individual	The individual person
Interpersonal	Relationships among team members within the clinic, and interactions between patients and staff
Institutional	The organisations involved, including the healthcare centre where the study was conducted and connected home care services and pharmacies.
Infrastructural	Refers to the social and cultural environment of the organisation, including laws, regulations, and educational system for staff, relating to healthcare, as well as changes in societal health patterns.

The findings are presented in a model based on Pawson’s main analytical framework [36] (Table 3). Examples of the data analysis, including the coding procedure, are shown in Table 2.

**Table 2:** Example of the data analysis process

Meaning unit	Code	Sub-category	Category	Main analytical category ^1^
‘Then the pharmacy’s list of current prescriptions is used instead of the medication list with prescribed medicines, patients may take double…incorrect doses (R2). I take five medicines daily… but the list does not state the timing… leaving me uncertain (R3).…the hardest part is the medication list. We use one… the pharmacy another, and we do not always provide our own (R4).I give the list to all my patients, but it does not always help (R2).Patients not recently hospitalised are unaware of our list and rely on the pharmacy’s prescription list (current prescriptions), which the pharmacy reviews with them (R4).Hospitals sometimes print lists without taking responsibility for paused medications (R2:2).We have two real official lists – Cosmic, Pascal, and then we have the pharmacy’s list (current prescriptions), and the patient’s own – and patients often believe the pharmacy list is the correct one (R4)’.	The medication list – a single document with clear information	Medication list – Clear and unambiguous prescribing information for daily intake of medications	Routines to support safe medication use	Infrastructural context/measure A single, national, clearly distinguishable medication list to be developed. Must explicitly serve as the basis for patient intake and should not be confused with other lists such as those for current prescriptions

^1^ Main analytical categories using Pawson’s theoretical framework, evaluating four dimensions of context: individual, interpersonal, institutional and infrastructural [36]

### Rigour/trustworthiness

To enhance the trustworthiness of the findings, investigator triangulation was used to ensure credibility [38]. All four researchers independently coded the data, and the codes were then compared, showing high agreement. Thereafter, the material was sorted into categories aligned with Pawson’s [36] analytical framework. Disagreements were discussed until a consensus and a common understanding of the categorisation was reached [38]. Reflexive discussions were held throughout the analytical process. To further increase credibility, data collection and analysis proceeded until no new information emerged (redundancy level).

In a PPI study, the aim is to capture diverse perspectives and practices, which can increase the value of the findings and contribute to changes in practice [26]. Using a theoretical framework for categorisation and presentation of data (Pawsons 2013 [36]) constitutes theory triangulation, further strengthening credibility [33, 38]. Confirmability was ensured by presenting examples of the coding and analysis process, including categories and sub-categories, illustrated by illuminative quotations. Dependability was supported by detailed descriptions of the research process. Participants’ characteristics and the study setting are provided to help the reader judge the transferability of the findings to other contexts or populations [38].

### Ethical considerations

The study was implemented in accordance with the Declaration of Helsinki (World Medical Association 2024) [39], and all procedures were approved by the Swedish Ethical Review Authority (Dnr 2020 − 00842). Written informed consent was obtained from all participants, outlining voluntary participation, the right to withdraw at any time without explanation and confidentiality, with data pseudonymised, handled and stored according to the regulations.

## Results

The support identified to develop a model promoting safe medication use in the home for persons with language difficulties that may pose a safety risk included seven components: routines to support safe medication use; continuity and risk assessment; support for language understanding; resources; information for patients and relatives about medication treatment and the prescribing system; staff training; and national measures. Further, support measures needed were identified across all four contexts of healthcare: individual, interpersonal, institutional and infrastructural. They were most prominent at the institutional and infrastructural levels, involving organisational development and the creation of guidelines and regulations by governing bodies. Fewer measures focused on the individual level, concerning the different professions involved and, to some extent, patients and relatives. Measures at the interpersonal level fell in between, and addressed everyday interactions and teamwork among healthcare and pharmacy staff. The components and support measures included in the service model are presented in Table 3.

**Table 3:** Support needed identified, with categories, subcategories, and proposed support measures, presented after sorted into the theoretical framework by Pawson (2013), evaluating four contextual dimensions of healthcare: individual, interpersonal, institutional and infrastructural

Support Needed	Support measures proposed at different levels in the healthcare system			
Category Sub-category	Context – main analytical categories			
	**Individual**	**Interpersonal**	**Institutional**	**Infrastructural**
**Routines to support safe medication use**				
Medication List – Clear and unambiguous information, used as the basis for the patient’s daily medication intake; distinct from prescriptions or pharmacy-issued lists.				Single, national, clearly distinguishable medication list to be developed. Must explicitly serve as the basis for medication intake and should not be confused with other lists such as those for current prescriptions.
Guidelines for prescribing procedures			Establish procedures to specify exact administration times on prescription documentation within the unit	Governing body: Collaborate with regional authorities (Regions and Pharmacy committee) to develop standardised procedures for prescribing medications, including exact administration times (e.g. 08:00).
Current medication list at visits - Always provided to patients during visits to the primary healthcare centre		Ensure medication list updated and printed at every physician visit. Nurses encouraged to distribute to patients		
Pharmacy-issued list of current prescriptions –provided upon patient request; not to be used as primary intake guide.		List of current prescriptions only issued by the pharmacy upon patient request. Not to be confused with the medication list		Recommendations from the governing body for pharmacy-issued lists handed out only on request from the patient
Pharmacists given the authority to update or remove outdated prescriptions				Professional regulations required from the governing body
Procedure for individualised care of foreign-born individuals with limited Swedish proficiency			Develop unit-level procedures to ensure culturally congruent care for patients experiencing language barriers	Governing body: Develop guidelines for individualised and culturally congruent care for foreign-born persons with limited Swedish proficiency.
Dose dispensing procedures		When initiating or adjusting medications in dose-dispensing system, changes must be clearly communicated to the patient and/or relatives.		
**Risk assessment and Continuity**				
Identify patients experiencing difficulties and assign a designated care contact	Enable self-booking and ensure patients are aware of a directly accessible designated contact person	The team/physician/nurse conducts risk assessment to identify patients at-risk, issuing notifications/ warnings and documenting in medical record as needed	Recruitment, job description, and planning for a directly accessible nurse, responsible for reviewing/updating/verifying the national medication list	Professional association to develop instructions for the ‘new’ specialist nurse function responsible for admissions and follow-up for this patient group
Involvement of relatives	Enable self‑booking and ensure awareness of a directly accessible designated contact person	Invite relatives to participate in visits or telephone consultations together with the patient, particularly during medication reviews. They should be encouraged to take notes.	Enable relatives to access up‑to‑date information in the patient’s medical record.	Develop digital solutions to allow relatives access patient information via the electronic health record system
Coordinated communication between different healthcare units and pharmacies	Staff must have/take the time to review information in existing inter‑unit communication systems, e.g. in transitions from hospital to home	Improve communication between pharmacy staff and primary care physicians through direct contact channels	Hold regular meetings across the care continuum, including pharmacies, healthcare centres, home healthcare services, and hospitals	Regular meetings between governing bodies at regional and municipal levels and pharmacy providers. Message‑transfer systems used for care transitions further developed for user‑friendliness and efficiency
Use of clinical pharmacist employed at the healthcare centre		Regular medication reviews conducted by the pharmacist, with physician contact as needed	Develop job description for pharmacists employed at the healthcare center; oversee recruitment/appointment of pharmacists	Governing body develops guidelines and professional regulations to ensure pharmacists conduct annual medication reviews
**Support for language understanding**				
Interpreter competence, education and training				Interpreter training should include proficiency in relevant language, knowledge of medical terminology, and skills in translation technique.
Use of interpreters		At end of each visit, set time for review/ clarification, e.g. go through the medication list, writing instructions on packaging, or accompanying the patient to the pharmacy if requested by the patient	Professional interpreter should be used, on site or via telephone, depending on need, in an undisturbed environment	
Bi/Multilingual staff in pharmacies, healthcare centres and homecare services		Possibility for translation support within the workgroup, provided by staff with diverse language skills	Policy to recruit bi-or multilingual staff when needed	
Relatives who speak Swedish	Swedish-speaking relative manages communication with healthcare services			
Use of procured digital systems for interpretation/translation	Mobile applications	Use the procured systems already available	Provide user training for existing systems, often not well known. Add language‑selection options to telephone answering system	
**Resources**				
Financial resources for extended consultation time with patients identified as being at risk				Address deficiencies in healthcare resources. Governing Body: financial, staffing, and structural resources. Ensuring access to competent staff, healthcare centres, and available care beds
Assistive tools: – Colour‑coding – Visual aids – Digitally accessible information	Use colour‑coding for medications	Promote use of visual aids and digitally accessible information within the workgroup	Develop/evaluate comprehensible digital assistive tools, recognising that technologies such as medication‑dispensing robots are not suitable for all patients. Promote implementation of digitally accessible information within the institution	Work for national standards for images and colour‑coding on medication packaging through governing body, alongside evidence‑based evaluation/development of assistive tools. Develop technical/digital support tools in collaboration with industry, such as enabling patients to contact or chat with healthcare services through translated multiple‑choice interfaces on mobile devices
**Information for patients and relatives to understand medication use**				
Medications: effects, generic substances, duration of treatment, and routine follow‑ups		Information and routine follow‑ups provided by nurses, physicians and clinical pharmacists	Patient education, including relatives – in group formats Information on medications, beliefs about illness and treatment, procurement and prescription processes, and associated costs. This education should be delivered in collaboration with primary care, pharmacies and home healthcare services.	
Medications and beliefs about illness/treatment		Information about illness, its chronicity, and treatment primarily provided by the physician, supported by nurses.		
Procurement and the medication prescribing system		Provide information and/or flowcharts explaining the procurement process and the medication prescribing system. Delivered by staff at healthcare centre and the pharmacy		
Costs for home care and medication administration		Provide information about costs related to home care services, home healthcare, medications, and dose‑dispensing services. Information should be given by staff in the healthcare centre, the pharmacy, home care and social care services.		
**Staff training**				
Training of healthcare professionals				Training in transcultural care and intercultural issues focused on beliefs about and use of medications, and the Swedish system for prescribing and procuring medications, delivered in an individualized manner.
			Workplace-based continuing professional development (CME) of personnel	Competence development – education and training of staff. CME integrated into the infrastructure — System developed for training of staff via universities and educational institutions
**National measures**				
Medication names: specify the active substance, or use simplified substance names to prevent confusion between synonymous brand names				Develop medication naming conventions specifying active substance or simplified substance name to prevent confusion. Mandated by the governing body and in alignment with WHO recommendations
Nationally unified electronic health record system and national medication list				Development of a national computerised system for patient medical records and a standardised medication list
National digitalised routine for bilingual medication prescribing, coordinated between healthcare centre and pharmacies			Provide information and training for physicians and pharmacy staff on use of digitalised system in the medical record, enabling multiple language options on prescriptions	Develop a secure, digitalised prescribing routine within the electronic health record that makes it ‘*easy to do the right thing’*, including automated language‑selection features
Language support for staff with limited Swedish proficiency			Workplace‑based language development projects	Language development initiatives led by the governing bodies of the healthcare system

### Routines to support safe medication use

The emerging picture is one in which the patient is at the centre, supported by routines for safe medication use and by infrastructural measures, including the development of a national single medication list, providing a clear and unambiguous record of prescriptions. Clear instructions on what to take, when, and including timing for each dose, should be provided. The list should not be able to be confused with other lists for current prescriptions. Furthermore, recommendations from the governing body should ensure that the list provided by the pharmacy for current prescriptions should only be handed out upon the patient’s request. Regulations should also allow pharmacists to remove or update outdated prescriptions.

The medication list should be updated and printed at every physician visit, and nurses within the interprofessional team should be encouraged to distribute the list to patients. Institutional routines for individualised and culturally appropriate care of foreign-born persons whose primary language is not Swedish should be developed, guided by infrastructure-based guidelines issued by the governing body.

*Having some sort of routine certainly helps… there is a routine… the medication list is supposed to be printed out during visits when medication changes are made… but this isn’t being followed… there could be a local routine… Remember to print out the medication list and ask the on-site interpreter to translate it for the patient after the visit*,* before the patient and the interpreter part ways.(R1) I also hand out… especially to those… who are on a lot of medications*,* and where you notice they need a medication list… [or] a new drug has been started. (R6)*

### Continuity and risk assessment

To ensure continuity and risk assessment in contact with those identified as having problems managing medications in the home, a designated care contact should be established. This role, clearly defined in a job description, should be filled by a specially trained individual or specialist nurse, with work organised and planned at the institutional level. The designated care contact should be directly accessible by telephone to the patient and their relatives and be responsible for reviewing, updating, and verifying the medication list. The role and responsibilities of the designated care contact should be regulated by infrastructural measures, such as instructions developed for staff via professional associations. Patients at risk should be identified within the interpersonal context by the care team, with the physician as the ultimate responsible. Risk profiles, including assessments of self-care ability, should be developed using instruments and notification systems informed by evidence-based research.

Relatives are an important resource in coordinated communication and may assist the patient by booking appointments with the designated care contact. They should be jointly invited with the patient by staff in the team (interpersonal context), particularly to participate in regular medication reviews, either in person or by telephone, and asked to take notes during visits. At the institutional level, relatives should be enabled to access up-to-date information in the patient’s medical record, supported by infrastructural measures to facilitate access to a digital e-health record.

Coordinated communication between different healthcare units and pharmacies is essential to maintain an unbroken care chain with the patient at the centre. Improved direct communication with the family doctor requires individual staff to allocate time to review information in existing messaging systems, e.g. transfers from the hospital to home. These processes should be supported by user-friendly logistical messaging systems, developed by governing bodies at the infrastructural level to simply transfer procedures. Regular participation in meetings between different units should take place at both the institutional and infrastructural levels.

Finally, a clinical pharmacist should be employed at the primary healthcare centre to conduct regular medication reviews in collaboration with the physician, as part of interpersonal collaboration. A clear job description for this role should be prepared at the institutional level, based on guidelines and regulations developed by the governing body at the infrastructural level to support annual medication reviews.

*Having a designated healthcare contact who actually has an overview of the entire list of medications*,* someone you can contact and ask questions. (R1)…it could be a nurse…someone who understands medications …(R6) A care coordinator… responsible for the patient’s entire course of treatment… could contact the doctor … now ‘Mr X’ has stopped taking his medication… so this could be flagged. (R1) …being able to channel inquiries… taking a closer look at how it works… gaining access via the geriatric risk profile… so that one can quickly highlight a patient’s needs… a dedicated point of contact… and a direct telephone line. (R3)…A drug risk score…high risk of treatment errors…I think that if you are going to solve the problem completely and permanently…something more is needed… we have special nurses for … patients with diabetes…those over 75 years. (R1)*

### Support for language understanding

For foreign-born persons with language difficulties, the main support for understanding the language is access to an interpreter. The interpreter should not only be skilled in translation techniques but also possess competence in medical terminology and pathology, ideally having completed specialised interpreter training supported at the infrastructural level. At the institutional level, a policy should ensure the recruitment of a professional interpreter available on site and working in an undisturbed environment. To make effective use of the interpreter, all visits involving members of the interprofessional team should include pre-planned time at the end together with the interpreter for activities such as reviewing the medication list, accompanying the patient to the pharmacy for interpretation, and explaining instructions on the medication packaging to facilitate the patient’s understanding.

The institutional policy should aim to employ bi-or multilingual staff within the work group or team, particularly in pharmacies, to provide translation support. Swedish-speaking relatives could also be used as a resource at the individual level to help patients communicate with staff in healthcare services. Finally, patients and staff should also receive training in using existing digital interpretation systems, including language selection on answering machines.

*It is extremely important that the interpreter has the necessary knowledge. They need to know a bit about medical terms… regarding diseases… and interpret exactly what the doctor is saying… not just about dialects*,* but the words…can be a big difference between the terms we use and others…. (R11)*

### Resources

Supporting this patient group requires infrastructural measures from the governing body, including financial resources to allow healthcare professionals additional time with persons identified as being at risk. Adequate support also depends on addressing limitations in healthcare resources, including financial, personnel and structural capacity, and ensuring access to competent and appropriate healthcare facilities.

Clear information about available resources and responsibilities, provided at both the institutional level and through interpersonal interactions, is necessary to help patients and healthcare professionals identify the appropriate point of care. Such clarity can prevent care being delivered at an inappropriate level and reduce situations in which patients are referred back-and-forth between different healthcare units without proper action, thereby avoiding inefficient use of resources.

To support safe medication use in the home, evidence-based research is needed to develop and evaluate different support tools. These may include visual support, such as national symbols and colour coding (e.g. indicating the time of dose on a dispenser), translated information/instructional materials; and technical/digital solutions for accessing healthcare, including translated multiple-choice functions; and mobile applications. Such tools should be developed under the leadership of governing bodies, in collaboration with industry.

At the institutional level, effective and user-friendly digital support tools should be developed and evaluated. Their use, including existing digital tools, should be promoted within the interpersonal care team, with support from the institution.

*There have to be resources also. The state must allocate extra resources to the primary healthcare centre… Do you want better care? Do you want fewer poisonings? Do you want better medication use? Then allocate resources. (R2)*

### Information for patients and relatives to understand medication treatment and the prescribing system

Patients and their relatives need to understand both their medication treatment and the system for prescribing and obtaining medications, including any related costs for care and support. Particular attention should be given to assessing and discussing individual beliefs about illness, chronic conditions and treatment.

Patients and their relatives should receive individual information from different staff at visits and routine follow-ups in the interpersonal context. In addition, participation in group-based patient education, which also includes relatives, should be offered at the institutional level. These activities should be carried out in cooperation among the physician, nurse, pharmacist, and home care staff. Thus, including the primary healthcare centre, pharmacy, and home care service.

*There is a lack of understanding of the prescription system and healthcare here in Sweden*,* a lack of knowledge about how the system is organised and where to turn for help. (R7)…If they could organise group meetings to explain the meaning of the medication list*,* current medications*,* how to think about medicines*,* and where to turn with questions…Explain synonymous medications … that is also a place where patients can meet …discuss with one another and get solutions and tips…. (R8)*

### Staff training

Infrastructural measures are needed to develop training for healthcare professionals and pharmacy staff and to establish a system for continuous medical education (CME), including workplace-based activities. Training should cover transcultural care and intercultural issues, focusing on beliefs about and use of medications, as well as the Swedish system for prescribing and dispensing medications, delivered using a person-centred, individualised approach.

*People with poorer compliance need support*,* and staff need to learn how to manage those visits…I organise visits differently for people with poor compliance and for foreign-born patients*,* making them more thorough. (R6)*

### National measures

Finally, a national Electronic Health Record system is needed, including a unified and unambiguous national medication list and a secure digitalised routine for prescribing medications with automatic language selection for bilingual support. This system should connect healthcare centres and pharmacies and serve as an infrastructural support measure. Collaboration with governing bodies and international organisations is needed to standardise medication names, specifying the active substance or a simplified substance name to prevent confusion between generic medications and reduce the risk of adverse drug events. The healthcare authorities should develop and implement language development projects at the institutional level for staff with limited Swedish proficiency.

*We cannot get a complete medication list digitally… And the national medication list. Everyone is just waiting for it. (R3)*

*The legislation should be that no medicine is allowed sold under anything other than the active substance name first. It should be Paracetamol-Alvedon*,* not just Alvedon. (R1)*

## Discussion

This study is unique in aiming to identify the support needed and to develop a support model through a co-creation participatory action research process involving end-users: foreign-born-persons, relatives and staff in healthcare and pharmacies. The aim was to promote safe medication use in the home and prevent medication errors among foreign-born persons identified as having language difficulties. Examining experiences from these different perspectives contributes to a comprehensive understanding of the complex process of medication use in the home.

The main results showed that a key measure to support safe medication use was establishing a routine that places the patient at the centre, supported by a single national, unambiguous medication list updated at every healthcare visit and clearly explaining what medication to take, when and why. For continuous support and risk assessment of patients identified as at risk, a designated care contact, a specially trained nurse who is directly accessible to patients and relatives, should coordinate the work of the interprofessional team. In collaboration with the pharmacist, this person would be responsible for regular medication reviews, updating the medication list, and co-ordinating communication across the healthcare chain. The support measures include all four contexts: individual, interpersonal, institutional and infrastructural. However, most measures were required at the institutional and infrastructural levels, involving organisational development and clearer guidelines/regulations from governing bodies. Fewer measures concerned the individual level, relating to different professions involved and, to some extent, patients and relatives. The interpersonal level fell in between, including everyday work and interactions among staff in healthcare and pharmacies.

The most important routine to support safe medication use was placing the patient at the centre, supported by a single, unambiguous national medication list, updated at every healthcare visit, explaining what medication to take, when and why. Confusion between different lists, such as the medication list and the pharmacy-issued list of current prescriptions, was identified as a threat to safe medication use. This issue emerged as a recurrent theme in previous studies involving foreign-born patients, relatives and healthcare and pharmacy staff, which formed the basis for this study [20–22], and represents a new finding. Others have discussed a lack of information, often related to discharge from hospital. For example, a review of ethnic minority groups, including migrants [12], identified misinterpretation of medication labels and misunderstanding of instructions. An interview study exploring Arabic-speaking migrant women’s experiences and perspectives on medication information [40] emphasised the need to provide written information at discharge, including a current medication list, detailing medications, their purpose and potential drug interactions. The healthcare system itself has been identified as a potential risk factor. A study exploring healthcare professionals’ perspectives on medication safety among older migrants with cognitive impairment and polypharmacy [5] highlighted overlaps in prescriptions, duplicate prescribing by general practitioners and specialist physicians, and non-updated medication lists. The way healthcare systems are configured shapes access to care and influences the quality of care provided [10]. Systemic factors play a key role, as the healthcare system itself functions as a social determinant of health – influencing and being influenced by – other social factors. Thus, this systemic problem should be addressed by developing a single, unambiguous medication list at the national level and by establishing institutional routines to ensure that updated medication lists are provided after every healthcare visit by members of the interprofessional team.

For continuous support and risk assessment of patients identified as at risk, a designated care contact, such as a specially trained nurse who is directly accessible to patients and relatives, should according to the participants coordinate the work of the team with the physician as the ultimate responsible and in collaboration with the pharmacist. This role would include responsibility for regular medication reviews to be made by the physician and the pharmacist, updating the medication list, and coordinating communication across the healthcare chain. The need for a specially trained nurse focusing on safe medication use in the home among foreign-born persons with language difficulties represents a new finding that has not previously been highlighted. However, specialist training for nurses has previously been developed in collaboration between professional associations and governing bodies for areas such as chronic disease management, e.g. diabetes care, and for care of elderly persons [41, 42]. The role involves providing specialist nursing knowledge through assessment of individual needs and coordinating care planning. To address the previously identified need for accessible care and adequate information for foreign-born persons with language difficulties and their relatives [20–22], this nurse should act as a designated contact person at the healthcare centre. The role should ensure accessibility for patients and relatives seeking support, while also coordinating communication with staff across the healthcare chain.

The team model itself is not new, but the extended team model for migrants, which also includes a pharmacist and relatives, is a new idea. Multi-morbidity and polypharmacy among older people pose challenges for health and social care systems. A multi-disciplinary, team-led comprehensive geriatric assessment of health and social care settings has therefore been described as a routine approach for assessing long-term conditions and optimising medication use [4]. The risk of these conditions is higher among migrants [2, 3]. In this model, early detection of persons at risk is central, and success depends on timely review and coordination so that the care plan responds to the patient’s needs and is developed through shared decision-making [4]. Pharmacists are ideally positioned to work alongside GPs [4]. In the present model, however, the pharmacist collaborates directly with the responsible physician and participates in regular medication reviews. To increase continuity, the pharmacist could be employed either by the primary healthcare centre or by the pharmacy, while working collaboratively within a seamless healthcare system, as emphasised from the patient perspective [14, 43]. Pharmacists play a crucial role in improving medication management. They are often the first point of contact in the healthcare system for newly arrived migrants and the last health professional to interact with patients before medications are used [16].

In this study, relatives supporting individuals with limited language proficiency in the home were identified as key actors and resources, responsible for organising prescriptions, interpreting information and mediating between healthcare providers and patients [22]. This aligns with findings from studies on older migrants with polypharmacy and cognitive impairment [5, 6]. Relatives also contribute to safety by voicing concerns to healthcare professionals and advocating for the patient [12]. In healthcare, close relatives are a highly valuable resource but are often underutilised [44]. Closer collaboration between relatives, patients and healthcare professionals is essential to improve medication safety [5, 12].

Active involvement of relatives and patients in both the healthcare process and the development of healthcare services is a key factor for efficient, high-quality care [12, 26], particularly in community-based settings [24, 25]. Primary care plays a central role in integrating services within the healthcare system. Coordinated communication between different healthcare and social care units throughout the healthcare chain is therefore needed [45] and should be seamless. As the first point of access to wider healthcare provision, primary care aims to deliver person-centred, continuous, co-ordinated and comprehensive care [10].

A prerequisite for developing efficient care, with a well-informed patient at the centre, is overcoming language barriers through the consistent use of professionally trained interpreters (skilled in both language and medical terminology), access to supportive information tools, and bi-or multilingual staff [14, 16, 19, 20–22, 40]. A new finding in this study was the need to plan dedicated time for a booked interpreter. Effective use involves setting aside time after each healthcare visit for the interpreter to answer questions and translate information, thereby extending the support needed for improved patient understanding.

Furthermore, overall support in terms of financial resources to allow extended time with at-risk persons, and digital tools to facilitate understanding of information, were identified as important. These measures contribute to a more efficient use of resources in a context of strained healthcare budgets [4, 25], ensuring that extra time and support can be provided where needed [4, 40].

According to Swedish legislation, including the Health and Medical Care Act (2017:30) [28] and the Patient Act (2014:821) [46], care must be person-centred and individualised. This implies that care planning should be based on an assessment of each individual’s beliefs and needs, and on interventions adapted to provide individualised and culturally congruent care, taking into account both lay and professional perspectives [47]. However, differences may exist between patients’ and relatives’ illness perceptions and the responsiveness of healthcare professionals to varying levels of health literacy [6, 47].

To enhance the understanding of medication and treatment as well as the Swedish prescribing and purchasing system, information should be provided to patients and their relatives both individually and in group formats. This work requires coordinated efforts among primary healthcare centres, home-care services, pharmacies and social services. Particular attention should be paid to individuals’ beliefs about illness, its chronicity and treatment regimens. Many migrants may be unfamiliar with chronic disease management, often coming from contexts where acute illnesses are more common and treated with short courses of therapy [19].

Another challenge is the risk of obtaining medications from other or home countries, where substances may not be equivalent to prescribed medications or may be taken in incorrect dosages [19]. Such practices increase the risk of adverse events [12] and must therefore be addressed within patient education. Education initiatives designed specifically for migrants—using culturally informed approaches—have shown promise. For example, a study targeting Arabic-speaking migrants demonstrated improved awareness of inappropriate medication use [18].

Targeted training for staff in both healthcare and pharmacy settings, along with a structured system for continuous medical education (CME) on health literacy, transcultural care, and intercultural understanding, is essential to ensure patients and relatives receive clear and comprehensible information. Such efforts are crucial for strengthening health literacy responsiveness among healthcare professionals [5, 6, 19, 47]. Additionally, several studies highlight the need for healthcare system–level changes to provide culturally competent care that effectively meets the needs of a multicultural society [12, 43, 48, 49].

National and international collaboration is needed to develop clear, consistent guidelines for medication lists, prescribing practices, job descriptions, and user-friendly tools to support implementation of this model in daily clinical work. Policy frameworks and organisations focused on improving healthcare quality increasingly prioritise migrants. A central challenge highlighted in recent research is how healthcare systems can ensure *culturally responsive and equitable* [49] patient safety for migrant populations [12]. Addressing this requires coordinated efforts to strengthen standards, improve communication, and reduce structural barriers to high-quality care. Such measures are essential to promote patient safety and ensure equitable care across diverse healthcare settings [12, 48, 49].

### Strengths and limitations

The main strength of this investigation is the broad representation of end-users, including foreign-born persons and relatives, as well as staff from healthcare (primary healthcare centre with connected home care) and pharmacies. All participants had been involved in previous studies on the use and management of medications in the home by foreign-born persons, identified by healthcare professionals as having language difficulties that may pose a safety risk [20–22]. Thus, patients, relatives, nurses (general and specialist), physicians and pharmacists active or working in the home, home care, primary healthcare centres and pharmacies, both foreign-and Swedish born, were represented and actively collaborated in the co-creation participatory action research process to develop the joint model for support [23]. The co-production process was conducted ‘with’ patients and the public rather than ‘about them’, following the ‘everyone included’ philosophy to ensure diversity of perspectives, mutual understanding and respect between researchers and lay persons, the core of PPI [26]. The potential influence of power dynamics on PPI is a well-known challenge [26, 34], but no signs of dominance were observed in the focus groups. Interactions were lively, active engagement was demonstrated, and all participants expressed themselves equally [32]. A key factor contributing to this positive climate was the involvement of skilled facilitators, experienced both in the field and in guiding discussions to ensure equitable participation, particularly among vulnerable and marginalised groups [33, 34], such as migrants. The lead facilitator was known to most participants, having interviewed them in previous studies [20–22], and invited them to take part in the present study. This familiarity helped build trust, enabled power sharing, and facilitated group interactions. This, in turn, promoted discussions, diversity of perspectives, and mutual understanding between researchers and lay representatives [26, 33, 34].

Another limitation is the potential for interviewer bias, as respondents might answer according to what they think the interviewer expects [38], particularly since many had met the same interviewer before. However, there was also a second facilitator acting as an observer of the group process, which decreased this risk and increased the credibility of the findings [32]. It is important to recognise that in co-production and action research guided by the PPI principle, the role of researchers and facilitators differs from conventional research. They are expected to be embedded in the process, embracing contradictions and exploring diverse experiences and practices across various realities, conducting research *with* participants instead of *on* them [23, 26, 33]. Thus, they should actively participate in discussions, promote power sharing and ensure diverse perspectives, instead of having an objective and distant role as researchers.

Recruitment proved challenging in this study due to several factors. Many potential participants were severely ill, had multimorbidity/polypharmacy, or had been hospitalised for adverse drug events. Others declined due to geographical reasons, hesitating to travel from remote areas, or because of last minute cancellations due to staff shortages, which reduced some focus groups to only two participants. Finally, the time elapsed since the previous study meant that many had left or changed workplaces due to the heavy workload in primary healthcare, making them impossible to reach. Specific groups, such as migrants, are often difficult for researchers to access because of their physical or social vulnerability or geographic location. Migrants can be particularly vulnerable because of their migration experiences and are therefore deemed hard-to-reach, making recruitment for research studies challenging [50]. This is particularly evident for individuals with a refugee background, as seen in previous studies [20–22].

The representativeness of the studied population may be questioned, given the various perspectives to be covered and the limited numbers of participants. However, the findings were consistent across participants, with rich and high-quality data, aligning with the previous studies forming the basis of this investigation [20–22]. There was broad representation of all end-users, including foreign-born patients and relatives, and healthcare and pharmacy staff, with diverse cultural (born abroad or in Sweden) and professional backgrounds (general and specialist nurses, physicians, pharmacists) and working in varied environments (home, home care, healthcare centres, pharmacies). The focus was on capturing a range of perspectives – disparity – to inform service improvement through co-production partnerships [23, 26, 33] and to develop a first prototype model [33] supporting safe medication use in the home for persons with language difficulties.

Although the model is based on experiences from real-world clinical healthcare discussed in focus-groups, including issues of practicality and implementation, further testing of its feasibility and sustainability, as well as further and development, is needed.

Finally, the question remains whether the model will work in settings where resources are limited, as establishing specialist care-coordination roles may require substantial funding and service redesign in many healthcare systems. However, organising care through multi-disciplinary teams with specialist functions and clearly defined roles has been recommended in the recent reform of primary health care in Sweden to develop high-quality and cost-effective healthcare [24, 25]. In some settings, mini-teams a physician and a nurse have been a well-functioning model for diabetes care for many years [41]. Thus, the model can be adapted to different local contexts. Adequate support for the safe use of medications in the home may prevent adverse drug events and ill-health and thus, costs, thereby reducing costs [12], and potentially freeing resources for the development of healthcare and service redesign.

## Conclusion

In conclusion, promoting safe medication use in the home for foreign-born persons with limited language proficiency requires a support model firmly anchored in primary healthcare. This model should include an extended interdisciplinary team, involving relatives and pharmacists in addition to primary healthcare and home care staff, coordinated by a designated contact person, specifically assigned and directly accessible to patients and relatives. This team should collaborate within a seamless and integrated healthcare system, ensuring a well-informed patient at the centre of care. Care planning should be based on an assessment of individual beliefs and needs, supported by clear guidelines, an unambiguous national medication list, defined roles and responsibilities, and systematically developed user-friendly tools.

The main implication for practice at the institutional and interpersonal levels is the need to critically review everyday workflows in primary healthcare centres, home-care services, and pharmacies. This review should identify how interdisciplinary collaboration can be organised and routines streamlined to free up time for essential activities. These include systematic risk assessments, medication reviews, standardised procedures to prevent confusion from parallel prescription documents, improved medication communication, and patient education involving relatives when relevant, while long-term infrastructural solutions are developed.

At the system level, in the infrastructural context, professional organisations, governing bodies, and policymakers play a central role in securing financial resources, developing national guidelines, and enacting supportive legislation. Particular investment is needed to develop interoperable electronic health records, including a national, unified medication list with automatic translation functions for use by primary care staff and pharmacies. Finally, sustained collaboration with universities and industry is also needed to support targeted staff training, research and innovations in technologies that prevent medication errors and promote safe medication use in the home.

## Data Availability

The datasets generated and/or analysed during this study are not publicly available to protect the integrity, anonymity and confidentiality of participants. Anonymised quotations supporting the results presented in the manuscript are included and additional data are available from the corresponding author upon reasonable request.
